# 

**Published:** 1889-12

**Authors:** 


### CONTENTS.

### *MISCELLANEOUS.*

| | PAGE |
|---|---|
| Announcement for 1890, | 409 |
| The Repertory, | 410 |
| Homœopathy of the Present as compared with that of Hahnemann. Dr. M. J. Buck, | 411 |
| A Broken Breast. Frank Kraft, M. D., | 414 |
| On Reporting Cases. S. L., | 417 |
| Clinical Medicine. G. W. Sherbino, M. D., | 419 |
| Clinical Cases. George Logan, M. D., | 420 |
| Therapeutics of Convulsions. H. P. Holmes, M. D., | 423 |
| Cases from Practice. Chas. L. Swift, M. D., | 435 |
| In Memoriam—Edward Bayard, M. D., | 437 |
| Resolutions of N. Y. Homœopathic Union, | 440 |
| In Memoriam—David Wilson, M. D., | 441 |
| In Memoriam—Henry Noah Martin, M. D., | 443 |
| Therapeutic Progress. H. H. Haralson, M. D., | 443 |
| The Hahnemann Homœopathic Hospital, of Rochester, N. Y., | 447 |
| Homœopathy Triumphant. L. L. Helt, M. D., | 449 |
| Dr. E. M. Hale's Cactaceæ, | 450 |
| A Case of Sunstroke. E. W. Berridge, M. D., | 451 |
| What Produces Death, | 452 |
| What is a Homœopath, | 453 |

### *BOOK NOTICES AND REVIEWS.*

| | |
|---|---|
| Transactions of Mississippi State Medical Association, | 454 |
| Relation of Homœopathy to Natural Science, | 454 |
| Photographic Illustrations of Skin Diseases, | 454 |
| Cremation of the Dead, | 454 |
| Transactions of the American Institute, | 454 |
| Therapeutics of Nervous Diseases with their Diagnosis and Pathology, | 455 |
| Physician's Pocket Day-book, Journal, and Ledger, | 455 |

### *NOTES AND NOTICES.*

| | |
|---|---|
| Erratum—Guernsey's Diphtheria Card—Blind from a Snake bite—Children's Homœopathic Hospital—A New Hahnemann Club—Surgical Journal—For Sale—One of Many—Epidemic Influenza—Removals, | 455 |

